# Non-occlusive mesenteric ischemia (NOMI): evaluation of 2D-perfusion angiography (2D-PA) for early treatment response assessment

**DOI:** 10.1007/s00261-020-02457-y

**Published:** 2020-02-26

**Authors:** Lena S. Becker, Klaus Stahl, Timo C. Meine, Christian von Falck, Bernhard C. Meyer, Cornelia L. A. Dewald, Nina Rittgerodt, Markus Busch, Sascha David, Frank Wacker, Jan B. Hinrichs

**Affiliations:** 1 Department of Diagnostic and Interventional Radiology, Hannover Medical School, Carl-Neuberg-Str. 1, 30625 Hannover, Germany; 2 Department of Gastroenterology, Hepatology and Endocrinology, Hannover Medical School, Hannover, Germany; 3 Department of Nephrology and Hypertension, Hannover Medical School, Hannover, Germany

**Keywords:** 2D-Perfusion angiography, Digital subtraction angiography, Non-occlusive mesenteric ischemia, Treatment efficacy

## Abstract

**Purpose:**

To evaluate the feasibility of 2D-perfusion angiography (2D-PA) for the analysis of intra-procedural treatment response after intra-arterial prostaglandin E1 therapy in patients with non-occlusive mesenteric ischemia (NOMI).

**Methods:**

Overall, 20 procedures in 18 NOMI patients were included in this retrospective case–control study. To evaluate intra-procedural splanchnic circulation changes, post-processing of digital subtraction angiography (DSA) series was performed. Regions of interest (ROIs) were placed in the superior mesenteric artery (SMA; reference), the portal vein (PV; ROI_PV_), as well as the aorta next to the origin of the SMA (ROI_Aorta_). Peak density (PD), time to peak (TTP), and area under the curve (AUC) were assessed, and parametric ratios ‘target ROI_PD, TTP, AUC_/reference ROI’ were computed and compared within treatment and control group. Additionally, a NOMI score was assessed pre- and post-treatment compared to 2D-PA.

**Results:**

Vasodilator therapy leads to a significant decrease of the 2D-PA-derived values PD_Aorta_ (*p *= 0.04) and AUC_Aorta_ (*p *= 0.03). These findings correlated with changes of the simplified NOMI score, both for overall (4 to 1, *p *< 0.0001) and for each category. Prostaglandin application caused a significant increase of the AUC_PV_ (*p *= 0.04) and TTP_PV_ was accelerated without reaching statistical significance (*p *= 0.13). When compared to a control group, all 2D-PA values in the NOMI group (pre- and post-intervention) differed significantly (*p* < 0.05) with longer TTP_Aorta/PV_ and lower AUC_Aorta/PV_ and PD _Aorta/PV_.

**Conclusion:**

2D-PA offers an objective approach to analyze immediate flow and perfusion changes following vasodilatory therapies of NOMI patients and may be a valuable tool for assessing treatment response.

## Introduction

Non-occlusive mesenteric ischemia (NOMI) was first described by Ende in 1958 in patients with severe heart failure [1]. Since then, NOMI is recognized as a substantial cause of acute mesenteric ischemia characterized by a combination of splanchnic vasoconstriction and the absence of embolic or atherosclerotic–thrombotic occlusion of the mesenteric arteries [2–4]. Risk factors for NOMI include cardiovascular and chronic kidney disease, administration of vasoconstrictive medications or drugs (e.g., digoxin, vasopressor therapy, cocaine, methamphetamine abuse), recent major surgery, sepsis, and shock [3, 4]. Overall, NOMI accounts for 5–15% of acute mesenteric ischemia cases [2, 3] and is associated with high mortality rates ranging between 50 and 93%, primarily due to its association with severe comorbidities [3, 5, 6]. Contrary to well-known symptoms of acute occlusive arterial mesenteric ischemia, clinical signs of NOMI are often unspecific [3, 4, 7]. Moreover, the symptoms may be masked due to intubation and sedation of the critically ill patients, potentially leading to a delayed diagnosis [3, 4, 7–11]. Laboratory tests (e.g., elevated serum lactate) in combination with increasing need of vasoconstrictive medications strengthen the clinical suspicion of non-occlusive mesenteric ischemia [3, 4]. To date, digital subtraction angiography (DSA) is the invasive gold standard to establish an imaging diagnosis by demonstrating signs like reduced blood flow, areas of narrowing, and spasms and consecutive irregularities of the mesenteric vessels (“string-of-beads” or “chain of lakes” appearance) [4, 10] although non-invasive computed tomography angiography is increasingly important for diagnosing NOMI [5, 12, 13].

Selective intra-arterial vasodilator therapy (e.g., prostaglandin E1, papaverine) is an effective treatment for NOMI [4, 5]. Monitoring of the vasodilatory effect on the splanchnic circulation, which is of utmost importance in the often critically ill patients, relies on subjective and non-standardized impressions of the interventional radiologist performing the procedure. NOMI scores proposed to predict outcome have not been evaluated for this task so far [7, 14]. In order to assess the presence of a therapeutic effect, quantify therapy response, and to avoid overtreatment in often severely hypotensive patients, a therapy response analysis tool would be valuable. Nowadays, 2D perfusion angiography (2D-PA) represents an emerging technique for the assessment and quantification of blood flow and tissue perfusion [15, 16]. It relies on the dedicated post-processing of standard DSA images and has been used in several organs and for different indications [17–20]. The use of 2D-PA for an objective analysis of the complex hemodynamic changes in NOMI and the ability to monitor treatment response immediately after application of vasodilators may be a valuable addition to improve local vasodilator therapy. Therefore, the purpose of this study is to evaluate the feasibility of 2D-PA measurements for the assessment of flow and perfusion changes following intra-arterial prostaglandin E1 infusions in patients diagnosed with NOMI.

## Methods

### Patients

This retrospective single-center observational case–control study, conducted in a tertiary care hospital from October 2018 to August 2019, was conducted in accordance with the ethical standards defined by the 1964 Declaration of Helsinki and its later amendments and was approved by the local ethics committee. Overall, 20 procedures in 18 consecutive patients were performed. Of these, one patient needed to be excluded due to severe motion artifacts, hindering an adequate 2D-PA analysis. Consequently, data of 19 procedures in 17 consecutive intensive care patients (see Table 1; 11 males, 6 females, mean age 60.9 ± 11 years) of different surgical and medical intensive care units with diagnosis of NOMI were included in our study. All patients received local intra-arterial prostaglandin E1 therapy in the interventional radiology suite. Diagnosis of NOMI was established by an interdisciplinary team following a standard work-up in our hospital [3]. Briefly, diagnosis was made up of a combination of clinical suspicion (decrease in oxygenation (PaO2/FiO2), pH, bicarbonate; increase of International Normalized Ratio (INR), lactate, bilirubin, leukocyte count, norepinephrine administration (NE), acute abdomen, or new onset of oliguria/anuria), reported radiographic signs of mesenteric ischemia on biphasic contrast-enhanced CT scan, and exclusion of thromboembolic causes [4, 12, 13]. Digital subtraction angiography (DSA) was subsequently performed. Findings of varying vessel diameters and narrowing of the SMA and its branches were used to confirm the diagnosis of NOMI. Subsequently, intra-arterial prostaglandin E1 infusion was started (20 μg, 10-min infusion) [3, 7, 12, 14, 21].

**Table 1:** Patients’ demographics

	NOMI cohort		Control group
N of procedures (total)	20		17
N of patients	18		17
Excluded	1		0
Included	17		17
Gender (*n*, %)			
Male	11 (64.7%)		10 (58.8%)
Female	6 (35.3%)		7 (42%)
Age (years) ± standard deviation	60.9 ± 11		59.06 ± 11.7
BMI (kg/m^2^)	27.5 ± 8.7		25.5 ± 6.7
Reason for admission (*n*, %)			
Surgical/interventional	4 (23,5%)		17 (100%)
Internal medicine	13 (76,5%)		
Lactate (mmol/L)	At time of intervention	12 h post- intervention	n.a.
	8.7 ± 3.4	6.90 ± 3.6	
Norepinephrine (*n*, %)	16 (84%)	16 (84%)	n.a.
Norepinephrine dose (μg/kg/min)	0.59 ± 0.33	0.58 ± 0.43	n.a.
pH	7.28 ± 0.12	7.3 ± 0.13	n.a.
Intubations (*n*, %)	15 intubations /19 procedures (79%)		n.a.
Comorbidities^a^	Acute kidney failure (7) Acute liver failure (5) Acute respiratory insufficiency (4) Atrial fibrillation (2) B cell lymphoma (1) COPD (1) Coronary heart disease (1) Diabetes mellitus (1) Dyslipidemia (2) Hypertension (2) Addison disease (1) Pancreatitis (2) Septic shock with multiorgan failure (16) Toxic megacolon, intestinal ischemia (3) Trauma (2)		Cancer (17) *Breast (1)* *CCC (4)* *HCC (7)* *Larynx (1)* *Pancreas (2)* *Ovarian (1)* *Uveal melanoma (2)* Dyslipidemia (4) Hypertension (5) Gastritis (3) Liver cirrhosis (8) Sarcoidosis (1) Thrombophilia (1)

*BMI* body mass index, *CCC* cholangiocarcinoma, *COPD* chronic obstructive pulmonary disease, *HCC* hepatocellular carcinoma, *N* number

^a^Diagnoses in alphabetical order, several diseases may occur concomitantly in one patient

As a control group, we analyzed 2D-PA data of 17 consecutive patients, who had porto-mesentericographies prior to local intra-arterial tumor treatment of the liver (male 10, female 7; age 59.06 ± 11.7 years).

### Digital subtraction angiography

All procedures were performed by board-certified, clinically experienced interventional radiologists using a monoplane, ceiling-mounted angiographic system (Artis Q®, Siemens Healthcare, Forchheim, Germany) or a monoplane, robotic-arm-mounted angiographic system (Artis pheno®, Siemens Healthcare, Forchheim, Germany).

Under local anesthesia and ultrasound guidance, a 4-F introducer sheath (Avanti + , Cordis, Waterloo, Belgium) was placed in the right or left common femoral artery. Afterwards, a diagnostic catheter was placed in the origin of the SMA (4F Cobra 2 or SideWinder 1, Terumo Radifocus® Glidecath®, Terumo Europe, Leuven, Belgium). Contrast medium (CM) was subsequently injected by a power injector (Accutron HP-D®, Medtron AG, Saarbruecken, Germany) in accordance with our standard NOMI protocol (flow rate 4 mL/s, 24 mL Iomeprol, 300 mgI/mL Imeron, Bracco Imaging, Konstanz, Germany). DSA images were acquired with a frame rate of four per second, until the portal vein was filled with contrast. DSA was performed before and after infusion of prostaglandin E1 (Alprostadil; UCB Pharma GmbH, Monheim, Germany) to assess early treatment response. 20 μg of prostaglandin E1 was infused over a duration of 10 min with the catheter securely positioned in the SMA. Sheath and catheter were left in place for subsequent local prostaglandin E1 infusion in the intensive care unit and removed upon clinical improvement (e.g., reduction of serum lactate levels, catecholamine doses by ≥ 20% and/or scoring of Sequential Organ Failure Assessment (SOFA)) or death.

### Image analysis and data evaluation

**Fig. 1:**
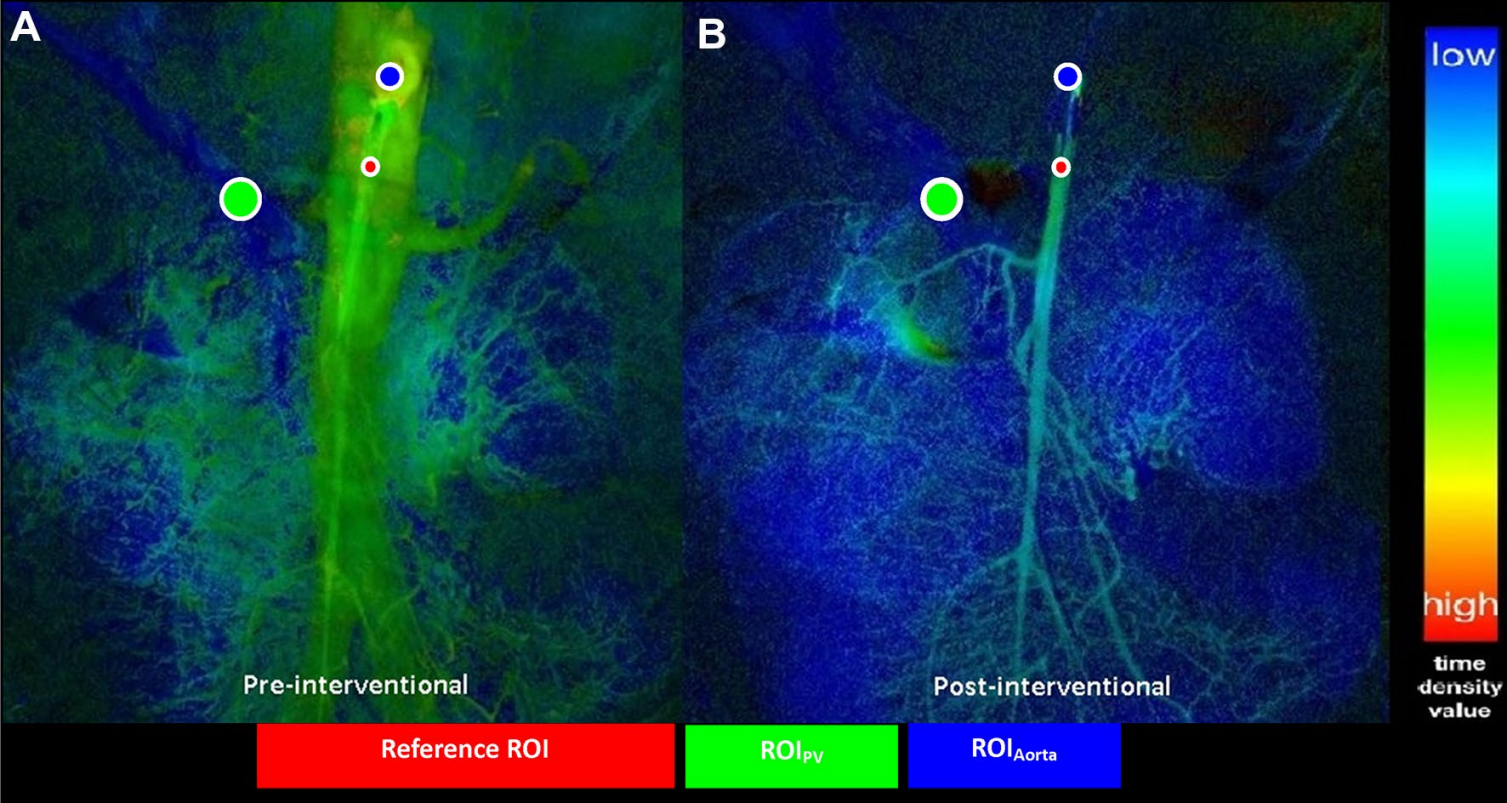
Definition of ROI placement pre- to post-intervention. ROI placement is shown in a 57-year-old female patient with NOMI, who underwent DSA and received prostaglandin therapy. **a**, **b** The reference ROI (red) is positioned in the SMA, adjacent to the catheter tip. Target ROI_PV_ (green) is placed within the portal vein, proximal to the hepatic bifurcation and target ROI_Aorta_ (blue) within the aorta, close to the origin of the SMA. The time density value is color-coded

Post-processing of the DSA runs was performed on a dedicated workstation (syngo X Workplace® VD20D, Siemens Healthcare). In consensus, two radiologists (J.B.H., L.S.B.) agreed upon ROI placement. A reference ROI was fitted to at least two-thirds of the vessel diameter and placed in the SMA next to the tip of the inserted diagnostic catheter and therewith at the location of CM influx. One target ROI was placed in the main stem of the portal vein (ROI_PV_), proximal to the bifurcation. Superimposition of bowel was avoided. A second target ROI was placed in the aorta (ROI_Aorta_) close to the origin of the SMA to detect contrast reflux at its origin (refer to Fig. 1). In the NOMI cohort the ROIs were then copied from the images acquired prior to prostaglandin infusion. In case of patient movement, the ROIs were manually moved to the respective locations on the post-interventional images. In the control group only one set of images was post-processed using size-related ROIs at the locations described in the NOMI group. Numeric density values for time to peak (TTP), peak density (PD), and the area under the time–density curve (AUC) were recorded. The ratios of the reference to the target ROI, i.e., TTP_PV_/TTP_Ref_, PD_PV_/PD_Ref_, AUC_PV_/AUC_Ref_, TTP_Aorta_/TTP_Ref_, PD_Aorta_/PD_Ref_, and AUC_Aorta_/AUC_Ref_ before and after vasodilatory therapy and in the control group were calculated. In accordance with Murray et al., PD is defined as the maximum density in the chosen ROI after CM administration, TTP is characterized as the time from the beginning of the angiographic run to the maximum density within the ROI, and AUC visualizes the density values within the ROI during the span of an angiographic run [22]. In addition, we assessed a previously published NOMI score [7, 14], that is comprised of three subjectively assessed categories: vessel morphology, aortal contrast reflux, and time to portal vein filling. We calculated the score before and after the intervention and analyzed its potential to monitor treatment response (see Table 2). Furthermore, serum lactate and NE levels were examined before and 12 h after catheter-based NOMI therapy.

**Table 2:** Simplified NOMI score according to Minko et al. [7]

Vessel morphology	
0	SMA trunk, branches and mesenteric arcades normal
1	SMA trunk normal, several SMA branches and/or mesenteric arcades slightly constricted SMA trunk slightly constricted, normal SMA brunches and mesenteric arcades SMA trunk, several SMA branches and/or mesenteric arcades slightly constricted
2	SMA trunk partly, multiple SMA branches and/or mesenteric arcades constricted x
3	SMA trunk, several SMA branches and/or mesenteric arcades slightly constricted
Contrast medium reflux into the aorta	
0	No contrast medium reflux into the aorta
1	Some contrast medium reflux into the aorta
2	Severe contrast medium reflux with complete aortogram
Time to portal vein filling	
0	≤ 8 s
1	< 8 to ≤ 12 s
2	> 12 s

### Statistical analysis

Descriptive statistical analysis of the patients’ demographics and angiographic data was performed. The results are presented as mean values with standard deviation. Comparisons between pre- and post-interventional 2D-PA parameters and with the control group were made using the pairwise Wilcoxon signed-rank test. Furthermore, a Spearman’s rank correlation was performed to compare 2D-PA values with the simplified NOMI score. A *p* value of < 0.05 was defined as significant. Statistical analyses were conducted using commercially available software (JMP Pro 13, SAS Institute, JMP Office Germany, Boeblingen, Germany).

## Results

After exclusion of one patient due to severe motion artifacts (combination of breathing and bowel movement), we analyzed a total of 19 procedures in 17 patients with the clinical- and imaging-based diagnosis of NOMI (male 11, female 6; age 60.9 ± 11 years) and, as a control group, 17 patients (male 10, female 7; age 59.06 ± 11.7 years), who underwent diagnostic angiography prior to local trans-arterial tumor therapy. For detailed demographics, clinical and interventional data, refer to Table 1.

The NOMI score changed significantly following prostaglandin E1 infusion (4 to 1, *p* < 0.0001; Table 2). Especially the subcategories “contrast medium reflux into the aorta” and “time to portal vein filling” decreased significantly after the intervention (*p *= 0.0075; *p *= 0.0002).

Reflux of contrast to the aorta, as reflected by changes in the PD and AUC values measured within the ROI_Aorta_, decreased significantly following vasodilator infusion (PD_Aorta_/PD_ref_: 0.74 ± 0.55 to 0.44 ± 0.38, *p *= 0.04; AUC_Aorta_/AUC_ref_: 0.71 ± 0.56 to 0.44 ± 0.38, *p *= 0.03), indicating a reduced backflow of blood to the aorta and an increase of blood flowing through the SMA. In accordance with a reduced backflow of blood to the aorta, contrast flow over time, as expressed by the AUC measured in the ROI_PV_, was significantly increased in the portal vein following vasodilatory therapy (0.69 ± 0.96 to 0.84 ± 0.62, *p *= 0.04). Time to peak values did not reach significance in the computed ROIs. Nevertheless, TTP was slightly accelerated in the portal vein following vasodilatory therapy (12.63 ± 3.6 to 11.49 ± 3.8, *p *= 0.13) which is in accordance with the change in “time to portal vein filling” assessed by the NOMI score. Except for the changes of PD_Aorta_ (*r* = 0.60; *p* = 0.006), none of the other 2D-PA values showed a correlation to changes in the NOMI score. NE levels, monitored at the time of intervention and 12 h later, were not significantly reduced. Lactate levels decreased by nearly 21% about 12 h following NOMI therapy, without reaching statistical significance (8.69 ± 3.44 to 6.9 ± 3.59; *p *= 0.06).

When comparing the 2D-PA values measured in the portal vein of our NOMI group to a normal control group, the mean PD and AUC values pre- and post-intervention were significantly higher than the corresponding PD and AUC values in the normal patient cohort (refer to Table 3; *p *< 0.05). Furthermore, the TTP measured in the portal vein of the normal cohort is significantly prolonged compared to the NOMI group, possibly reflecting the hyperdynamic circulation state of the NOMI patients (*p *< 0.01). Comparable results were found in terms of the aortic ROI when assessing the backflow of contrast media to the aorta, with a significantly shorter TTP 11.54 s to 8.52 (pre-intervention vs. norm; *p *= 0.007) and 8.46 (post-intervention vs. norm; *p *= 0.01). Accordingly, the PD and AUC in the aorta were significantly higher in the NOMI cohort (pre- and post-interventional) when compared to the control group (*p *< 0.01). Nevertheless, even in the control group, backflow of contrast media into the aorta could be detected (*n* = 7).

**Table 3:** Results of the control group

	Control group	NOMI group pre- intervention	NOMI group post-intervention
		*p* value	*p* value
*PD*			
PD_PV_norm_			
PD_Aorta_norm_	0.40 ± 0.19	0.04	0.005
*TTP*	0.21 ± 0.15	0.002	0.04
TTP_PV_norm_	15.97 ± 2.2	0.0002	< 0.0001
TTP_Aorta_norm_	11.54 ± 4.6	0.007	0.01
*AUC*			
AUC_PV_norm_	0.26 ± 0.1	0.06	0.005
AUC_Aorta_norm_	0.19 ± 0.07	0.001	0.033

Mean values ± standard deviation between pre- and post-intervention

*AUC* area under curve, *NOMI* non-occlusive mesenteric ischemia, *PD* peak density, *PV* portal vein, *TTP* time to peak, *Ref* reference

## Discussion

Previous studies have shown that NOMI is a complex disease with high morbidity and mortality rates [3, 5]. By combining diagnostic and therapeutic opportunities, angiography remains the modality of choice for disease diagnosis and offers the opportunity for immediate treatment [2, 5]. Our current study shows that 2D-PA is clinically feasible and an applicable technique to analyze and quantify perfusion changes immediately after vasodilatory treatment of NOMI. Moreover, 2D-PA analysis manages to convey a multitude of information about vessel morphology, color-coded hemodynamics, and semi-quantitative data at a single glance. Therefore, 2D-PA has the potential to simplify not only the diagnosis but also the treatment response analysis in NOMI patients. We demonstrated a significant decrease of aortal CM reflux following interventional NOMI therapy, as reflected by changes in PD and AUC values measured in the corresponding ROI_Aorta_. Combined with a reduced backflow of CM to the aorta, a higher CM flow over time could be demonstrated in the portal vein as expressed by an increased AUC value measured in ROI_PV_. While TTP values did not reach statistical significance, the tendency of changes of TTP in both target ROIs supported our overall conclusion by showing a decrease in the time to maximal contrast in the portal vein and an increase in the ROI_Aorta_. The comparison of patients with NOMI to a control cohort without NOMI showed a distinctly prolonged TTP_PV_ and TTP_Aorta_ in the control cohort. Furthermore, the values for PD and AUC in both target ROIs were significantly lower compared to pre- and post-interventional measurements in the NOMI cohort (for details refer to Table 4). In addition, we analyzed lactate levels at 12 h pre- and post-intervention according to the study by Stahl et al. [3]. Despite a positive trend, the 12-h lactate level reduction did not reach statistical significance (*p *= 0.06). One reason for the non-significant lactate decrease might be our small sample size. Other reasons include the 12-h time period for monitoring and/or other potential causes for a higher lactate level (e.g., liver failure). Nevertheless, Klotz et al. suggest that lactate levels as biomarkers for NOMI severity and post-interventional treatment response might be a useful indicator, but are lastly unspecific [21]. They also described parallels between angiographically severe NOMI and the degree of lactate increase without significant correlations between these values [21].

**Table 4:** Results of the NOMI cohort

	Pre-interventional	Post-interventional	Mean difference (%)	*p* value
Simplified NOMI score	4 [3; 5]	1 [0; 2]	− 3 (75%)	< 0.0001
*PD*				
PD_PV_/PD_Ref_	0.86 ± 0.94	1.07 ± 0.69	+ 0.21 (24.4%)	0.10
PD _Aorta_/PD_Ref_	0.74 ± 0.55	0.44 ± 0.38	− 0.30 (40.5%)	0.04
*TTP*				
TTP_PV_/TTP_Ref_	12.63 ± 3.6	11.49 ± 3.8	− 1.14 (9.0%)	0.13
TTP_Aorta_/TTP_Ref_	8.24 ± 3.52	8.64 ± 4.95	+ 0.40 (4.9%)	0.73
*AUC*				
AUC_PV_/AUC_Ref_	0.69 ± 0.96	0.84 ± 0.62	+ 0.15 (21.7%)	0.04
AUC_Aorta_ AUC_Ref_	0.71 ± 0.56	0.44 ± 0.38	− 0.27 (38.0%)	0.03
Lactate level (intervention/12h post-intervention)	8.69 ± 3.44	6.90 ± 3.59	− 1.79 (20.6%)	0.06

Mean values ± standard deviation and (% difference) between pre- and post-intervention

*AUC* area under curve, *NOMI* non-occlusive mesenteric ischemia, *PD* peak density, *PV* portal vein, *TTP* time to peak, *Ref* reference

The highly subjective NOMI score ranging from 0 to 7 with its subcategories “aortal reflux, time to portal vein filling and vessel morphology” proved significant in our cohort, when analyzing the response to vasodilatory therapy. However, the comparison of the 2D-PA values with categories of the simplified NOMI score did not prove to be significant, with the exception of peak density measured in the aorta (*r* = 0.60; *p* = 0.006). According to Minko et al., who evaluated the outcome based on the NOMI score, ≥ 3.5 is associated with increased peri-operative mortality and elevated serum lactate and thus indicates severe NOMI [7]. With an overall score of 4, our cohort was slightly above this threshold. However, there was still a disparity between the clinical indicators for NOMI and the computed score: even with extremely high clinical suspicion of NOMI, the median score was 3.8. For the categories of the NOMI score, Minko et al. published *ĸ* values comparing radiologists, an intensive care physician and a medical student [14]. For “vessel morphology” they reported moderate agreement between two radiologists(*ĸ* = 0.51) and poor agreement (*ĸ* = 0.02) between radiologist and medical student, indicating a high operator dependence of this parameter [14]. “Time to portal vein filling” showed a *ĸ* value of 0.42 between the radiologist and the intensive care physician, also indicating only moderate agreement between highly trained physicians [14]. The category “aortal reflux” had the highest *ĸ* value between all investigators (*ĸ* = 0.82–0.63) [14]. However, in our study, contrast reflux to the aorta could be detected even in several subjects of our normal cohort, indicating this finding not to be NOMI-specific. Taking these findings into account, the categories of the NOMI score are constrained by high inter- and intraobserver variability. This underlines the need for a more objective analysis tool and underscores the potential value of the 2D-PA measurements presented here.

Comparable to the radiographic signs suggestive of NOMI, the pre-interventional 2D-PA images showed aortal CM reflux of varying severity, a prolonged time to portal vein filling, and narrowing or spasm of the peripherally rarefied mesenteric arcades. After prostaglandin infusion, significantly less aortal CM reflux, pathologic vessel morphology signs and reduced time to portal vein filling could be visualized, indicating a positive vasodilating effect of locally administered prostaglandin E1. The subjective and highly variable impression and assessment of the radiographic signs of NOMI might benefit from the addition of objectively computed parameters such as the 2D-PA time density values analyzed in this study. As described above, 2D-PA images were computed and identical ROIs were placed in strategic positions in the proximal SMA (reference) as well as in the portal vein prior to the bifurcation (target) and the aorta (target), close to the origin of the SMA. These targets were chosen in analogy to the NOMI score, to measure CM flow, avoid potential superimposition of intestines, and to evaluate and compare perfusion ratios of the parameters TTP, AUC and PD in the NOMI cohort and a control group. Our measurement of the CM reflux in the aorta is comparable to the aortal CM reflux of the NOMI score, which was statistically the most robust category in a study by Minko et al. [7], and our results were comparable in this regard. However, 2D-PA parameters measured in the portal vein differ substantially from the subjective detection of “first contrast agent arrival in the portal vein” assessed by the NOMI score. Novel information is gained from the evaluation of AUC_PV_: by measuring the post-interventionally increased flow of CM through the portal vein over time and thus having an indicator of not only a faster but also a higher amount of blood flowing through the SMA territory.

To date, 2D-PA is a subject of increasing scientific interest with the potential to objectively assess flow and perfusion parameters on the basis of assigning density values to pre-defined ROIs, comparable to previously described magnetic resonance imaging (MRI) and CT techniques [23, 24]. Contrary to CT or MRI, angiographic 2D-PA is available during the intervention and therefore permits arterial inflow monitoring at table side [18, 22, 25]. Therefore, flow and perfusion parameters potentially influencing therapeutic strategies become available during the intervention. Per frame, mean density values can be computed and employed for the calculation of the maximal density values, flow rates and flow times [22, 25]. As mentioned earlier, 2D-PA image generation and analysis does not need additional CM application or additional radiation exposure due to supplemental DSA runs. The images contain clustered information pertaining hemodynamics and vessel morphology in one single picture and may be of benefit in the diagnosis and treatment process of NOMI. However, a precondition for widespread use is an integrated image analysis and presentation option, facilitated by an interactive, semi-/automated workflow from the table side.

In the control group, we observed a significantly longer TTP in the aorta as well as PV and decreased PD and AUC values in both target ROIs, when compared to the NOMI cohort pre- and post-intervention. The mean time to portal vein filling in patients without symptoms of mesenteric ischemia was recorded at nearly 16 s, compared to a mean of 12 s in the NOMI cohort. The particular hyperdynamic circulation of critically ill patients might be a possible explanation for this finding. Due to high amounts of internal neurohormonal mediators, in addition to externally administrated catecholamines, the splanchnic and peripheral circulation are short-circuited in favor of cardiac and cerebral blood flow and therewith, a lower amount of blood flows through the SMA. Nevertheless, a partial compensation is achieved with higher pulse and stroke volumes and thus a faster passaging of a reduced blood flow through the splanchnic circulation potentially causing a faster filling time of the portal vein in NOMI patients compared to a normal cohort [1, 2, 4].

**Fig. 2:**
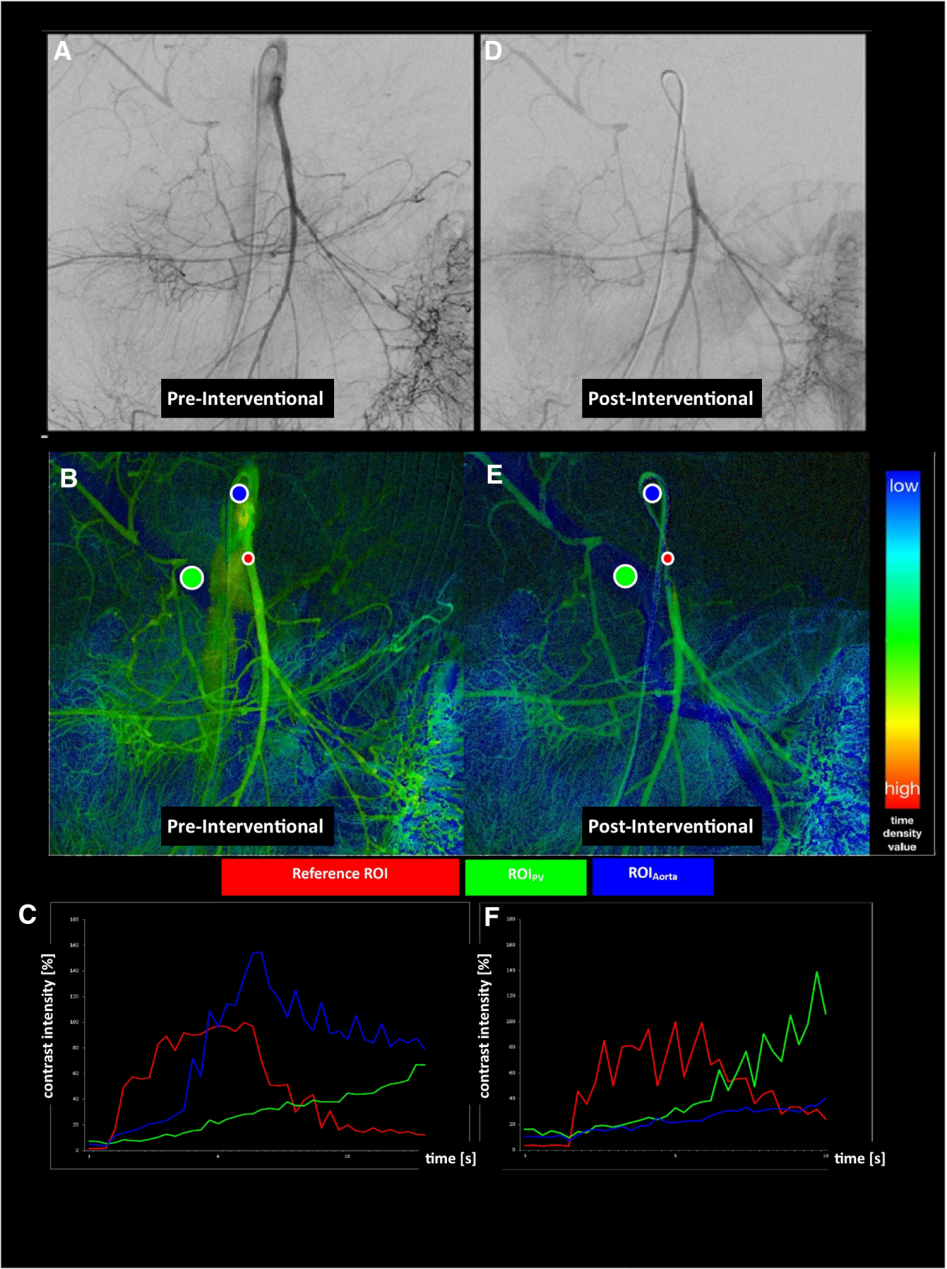
In this 52-year-old female patient with NOMI, intervention with prostaglandin E1 therapy in the superior mesenteric artery is demonstrated. Note the differences in vessel morphology, contrast enhancement, and aortal CM reflux. **a** Pre-interventionally, the vessels in this angiography series are smaller in diameter and peripherally rarefied. There are higher time density values in the dependent areas on the color-coded 2D-perfusion angiography (**b**) and a longer time to peak for ROI_PV_ (**c**). **d** After prostaglandin infusion, aortal CM reflux is no longer detectable, leading to decreased time density values within the corresponding ROI_Aorta_ (**e, f** blue ROI and blue line) and increased contrast density values in the portal vein (**e**, **f** green ROI and green line)

The susceptibility of 2D-PA measurements to movement has been previously described for endovascular treatment of peripheral arterial disease and tumor perfusion analysis after conventional transarterial chemoembolization (cTACE) [15, 26, 27]. Subsequent movement artifacts can cause inaccurate measurements as well as misinterpretations of the perfusion parameters, and should be avoided [15, 26, 27]. By providing clear breathing instructions for sentient patients and by utilizing controlled breathing conditions in anesthetized patients, motion artifacts due to ventilation might prove controllable to a certain level. Nevertheless, even in patients under general anesthesia, movement due to ventilation may occur. Another factor causing movement artifacts and impairing 2D-PA analysis is the motility of the intestines. To avoid potential inaccuracies by intestinal movement, we chose ROI locations within the portal vein and the aorta without superimposition of intestinal loops. Even though the administration of drugs causing intestinal relaxation may potentially improve image quality, they might on the other hand worsen the hemodynamic condition of NOMI patients. Overall, we needed to exclude only one patient from our study due to severe motion artifacts (see Fig. 2).

 Our study has several limitations. The number of patients in our retrospective study at a single medical center was small. Furthermore, the control group consisted of individuals with liver tumors. Although unlikely, the abovementioned underlying disease might influence the splanchnic circulation. In addition, our target ROIs did not encompass the entire lumen of the portal vein or capture the entire reflux to the aorta, which could influence the measurements. Another limitation is the absence of a gold standard for the 2D-PA measurements during NOMI interventions. To address this issue, we also evaluated the simplified NOMI score pre- and post-treatment. The primary focus of our study was the feasibility of 2D-PA images to evaluate treatment response in NOMI patients—a longitudinal follow-up and a survival analysis were therefore not included. Further studies, preferably larger, prospective multicenter studies with long-term follow-up, are needed to determine cut-off values generated from 2D-PA for a more precise categorization of NOMI, an adequate treatment response, and to confirm our data. For this reason, we have initiated a subsequent prospective study to further evaluate the value of 2D-PA for NOMI diagnosis and therapy response.

## Conclusion

2D-PA offers an objective approach to analyze immediate flow and perfusion changes following vasodilatory therapies of NOMI patients and may be a valuable tool to monitor treatment response.

## Abbreviations

2D-PA: 2D-Perfusion angiography
AUC: Area under the curve
BMI: Body mass index
CM: Contrast medium
CT: Computed tomography
cTACE: Conventional transarterial chemoembolization
DSA: Digital subtraction angiography
INR: International Normalized Ratio
MRI: Magnetic resonance imaging
N: Number
NE: Norepinephrine
NOMI: Non-occlusive mesenteric ischemia
PD: Peak density
PV: Portal vein
PaO_2_/FiO_2_: Ratio of arterial oxygen partial pressure to fractional inspired oxygen
ROI: Region of interest
ROI_Ref_: Reference region of interest
ROI_PV_: Region of interest in the portal vein
ROI_Aorta_: Region of interest in the aorta
Sec: Seconds
SD: Standard deviation
SMA: Superior mesenteric artery
SOFA: Sequential Organ Failure Assessment
TTP: Time to peak
